# Daily 800 mg versus 600 mg Efavirenz for HIV Patients Treating Tuberculosis with a Rifampicin-Based Regimen: An Open Label Randomized Controlled Trial

**DOI:** 10.1155/2018/9231835

**Published:** 2018-12-25

**Authors:** Mariana S. Xavier, Anete Trajman, Carolina A. S. Schmaltz, Flavia M. Sant'anna, Ivan R. Maia, David J. Hadad, Pedro Emmanuel A. A. do Brasil, Valeria Rolla

**Affiliations:** ^1^Plataforma de Pesquisa Clínica da Vice-Presidência de Pesquisa e Coleções Biológicas/Fiocruz, Brazil; ^2^Programa de Pós-Graduação em Clínica Médica, Universidade Federal do Rio de Janeiro, Brazil; ^3^McGill University, Canada; ^4^Laboratório de Pesquisa Clínica em Micobacterioses do Instituto Nacional de Infectologia Evandro Chagas/Fiocruz, Brazil; ^5^Núcleo de Doenças Infecciosas, Universidade Federal do Espírito Santo, Brazil; ^6^Laboratório de Pesquisa Clínica em Imunizações e Vigilância do Instituto Nacional de Infectologia Evandro Chagas/Fiocruz, Brazil

## Abstract

**Objectives:**

Pharmacokinetics studies recommend increasing efavirenz dosage in tuberculosis/HIV patients using rifampicin. We aimed to evaluate efficacy and safety of 600 versus 800 mg of efavirenz in tuberculosis/HIV patients using rifampicin.

**Design:**

We conducted an open label, multicentre, randomized trial from 2006 to 2012. The primary outcome was the proportion of undetectable viral load (HIV-VL) within six months. Secondary outcomes were time to achieve primary endpoint, trajectories of HIV-VL, proportion of any adverse events (AE), proportion of severe and serious AE (SSAE), and time to treatment interruption due to SSAE.

**Methods:**

Efavirenz-naïve patients were randomized 30 days after rifampicin-containing regimens initiation to receive 600 (comparison arm) or 800 mg (intervention arm) efavirenz-based regimens and followed-up for 180 days.

**Results:**

Sixty-five and 67 participants were respectively included in the comparison and intervention arms with 64.6% (52.5%-65.1%) and 62.7% (50.7%-73.3%) attaining undetectable HIV-VL in six months. Median time to attain undetectable HIV-VL was 70 days in both arms, with HIV-VL overlapping trajectories during follow-up. Cough, acne, and dizziness were more frequent in the intervention arm. SSAE were observed in 19.1% (13.8%-25.8%) and 25.0% (18.9%-33.2%), respectively. Survival curves up to the first SSAE-attributed treatment interruption were similar. None of the differences were statistically significant.

**Conclusion:**

Efficacy of efavirenz was similar regardless of dosage. Differences regarding safety occurred as mild and transient events, which did not interfere with treatment. Similar efficacy and safety (SSAE) and lower tolerance (minor AE) in the intervention group favour the use of 600 mg efavirenz in patients using rifampicin.

## 1. Introduction

Tuberculosis (TB) in people living with HIV/aids (TB-HIV) is a global public health problem, especially in places with high TB incidence, such as Brazil [1]. The World Health Organization (WHO) estimated 1.2 million (11%) people living with HIV worldwide in 2015 [2]. TB treatment for TB-HIV patients is the standard RHZE regimen: rifampicin (RIF), isoniazid, pyrazinamide, and ethambutol [3]. Rifabutin may replace RIF in selected cases [3, 4].

Efavirenz (EFV) is a potent nonnucleoside reverse transcriptase inhibitor (NNRTI) which is part of the first-line treatment for HIV infection in Brazil and other TB high-burden countries [5, 6] because of its low cost and better tolerance when compared to protease inhibitors (PI). EFV is metabolised by CYP3A/CYP2B6, while RIF is a strong CYP3A as well as a moderate CYP2B6 inducer [7]. Therefore, the concomitant use of RIF and EFV may result in an additive effect to induce CYP2B6 enzymes, which in turn reduces EFV mean peak concentration, trough concentration, and area under the concentration-time curve over the administration interval which decreased 24%, 25%, and 22%, respectively, in patients using both drugs [8]. EFV's bioavailability is decreased when used in concomitance with RIF [8–10], which may increase the risk of viral resistance. The phenomenon may be more evident in patients weighting over 50 kg [11].

Because of this interaction, some authors argue that the EFV dose should be increased from the standard 600 mg to 800 mg daily when used in concomitance with RIF [7]. Moreover, based on these pharmacokinetics studies ([8–10], the manufacturer has recommended the use of 800 mg for patients using RIF concomitantly [12]. However, clinical evidence regarding the efficacy and safety of 600 versus 800 mg is scarce, thus guidelines [2, 3, 6, 13] generally recommend the use of 600 mg. The aim of the present study was to evaluate the efficacy, safety, and tolerance of EFV 800 mg/day compared to EFV 600 mg/day in patients with TB-HIV treated with RIF-based regimens, in order to build stronger evidence that would justify recommending higher doses of EFV in spite of the potential risk for more adverse events (AE).

## 2. Methods

### 2.1. Study Design and Settings

This study consisted of an open label parallel randomized clinical trial for patients with TB-HIV using RIF-containing regimens in three sites in Brazil:* Instituto Nacional de Infectologia *Evandro* Chagas* (INI/Fiocruz),* Núcleo de Doenças Infecciosas* from* Universidade Federal do Espírito Santo* (NDI/UFES), and* Hospital Santa Casa da Misericórdia*, in Rio de Janeiro (SCM). Patients were randomized to receive either EFV 600 mg (EFV600 – comparison arm) or EFV 800 mg (EFV800 – intervention arm) daily. The study was open label for safety and tolerance (for patients and physicians), but laboratory technicians, who analysed the main outcomes, did not know the allocation. The protocol was conducted from April 2006 to March 2012.

### 2.2. Participants

The study population consisted of patients (a) aged 18 years or older, (b) infected with HIV as confirmed by two positive ELISA tests or one ELISA and another confirmatory test or HIV viral load (HIV-VL), (c) diagnosed with TB (defined by clinical signs and symptoms of TB for temporary eligibility until confirmation by identification of* Mycobacterium tuberculosis* in culture or, when not confirmed by culture, if symptoms subsided after antituberculous therapy provided that other opportunistic diseases were excluded), (d) with life expectancy greater than one year (Karnofsky score ≥ 70%), (e) not using other drugs than RIF with significant EFV interaction, (f) naïve for any NNRTI or protease inhibitor (PI), (g) with no history (current or past) of treatment with more than two nucleoside reverse transcriptase inhibitors (NRTI), and (h) voluntarily signed the informed consent (IC) form.

Patients were excluded if (a) antiretroviral (ARV) treatment was not indicated at the time of signing the IC form, (b) they had a history of hypersensitivity, intolerance, or resistance to RIF, (c) they had any psychiatric disorder, (d) one or more of the following laboratory abnormalities were present: liver transaminases ≥ 5-fold the upper level of normality or total bilirubin ≥ 1.5 mg/dL, or (g) they were pregnant.

Patients with low adherence to ARV and baseline resistance to RIF or EFV diagnosed during follow-up were excluded from the per protocol analysis but were kept for the intention-to-treat and safety analyses until discontinuation.

### 2.3. Intervention

All participants received first-line TB treatment according to the Brazilian guidelines [14] for patients over 45 kg at the time of inclusion: an intensive phase consisting of two months of RIF (600 mg/day fasting) + isoniazid (400 mg/day) in fixed-dose combination and pyrazinamide (2 g) followed by a four-month continuation phase with RIF and isoniazid in fixed-dose combination at the same doses for those included until 2010. Since December 2010, Brazilian guidelines incorporated the WHO-recommended RHZE regimen with RIF 600 mg, isoniazid 300 mg, pyrazinamide 1500 mg, and ethambutol 1100 mg in fixed-dose combination for two months, followed by four months of RIF 600 mg + isoniazid 300 mg in fixed-dose combination, for both new and retreatment cases until drug-susceptibility tests are available [13]. For patients weighting less than 45kg, RIF dose was adjusted (10mg/kg) for both regimens.

All participants received background optimised ARV therapy, which consisted of two NRTI plus EFV600 or EFV800. The preferred initial NRTI were zidovudine + lamivudine in fixed-dose combination or lamivudine and tenofovir.

Patients in the comparison group received one pill of 600 mg EFV, and patients in the intervention group received one 600 mg plus one 200 mg pill. A complementary placebo pill was not available; thus, blinding (patient and physician/nurse) was not possible.

### 2.4. Outcomes

Efficacy was evaluated through HIV-VL, safety through severe and serious AE (SSAE), and tolerance through minor AE. The primary endpoint was the proportion of undetectable HIV-VL (<80 copies/ml) on day 180. Secondary endpoints were (a) time to achieve undetectable HIV-VL, (b) HIV-VL (log⁡10) trajectory over time in all trial population and subgroups (initial HIV-VL log⁡10 > 5, initial HIV-VL log⁡10 < 5 and patients above 50 kg), (c) proportion of any reported AE, (d) proportion of SSAE, according to WHO Adverse Reaction Terminology (WHO-ART) [15], and (e) time to discontinuation of treatment (ARV or RIF) attributable to SSAE.

We defined AE as any unwanted medical event that started after the onset of the concomitant use of EFV and RIF, i.e., after randomization. AE were classified according to the AIDS Clinical Trial Group (ACTG) grading [16].

### 2.5. Randomization

The allocation sequence was computer-generated with simple random numbers and performed by the pharmacy of the coordinating centre, INI/Fiocruz.

### 2.6. Procedures

**Figure 1:**
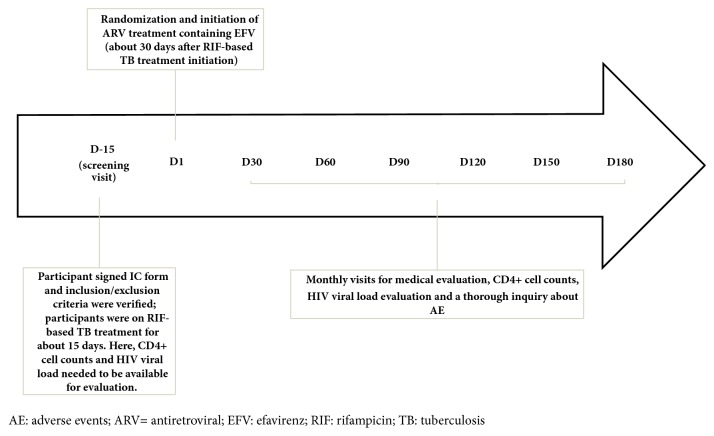
Trial timeline.

Timeline of all procedures is represented in Figure 1. When patients with suspected TB were evaluated, anamnesis and physical examination were conducted and chest radiograph, smear microscopy, sputum culture with drug-susceptibility test, blood culture, tissue biopsies with histopathological evaluation when applicable, whole blood cell count (WBC), liver and renal function tests, and serology (for HIV, hepatitis B and C, toxoplasmosis, and syphilis) were performed. For those who were HIV-positive (potentially eligible), CD4+ cell counts and HIV-VL were performed to check the eligibility according to Brazilian guidelines. Those who started RIF, isoniazid and pyrazinamide (regardless of dose and of ethambutol use, i.e., both according to previous [14] and current [13] national guidelines) were evaluated during a visit 15 days after TB treatment initiation (D-15, the screening visit). Patients were invited to participate in the trial and those who agreed signed the IC form. After the IC process, anamnesis and a complete physical examination were undertaken and the Karnofsky score was calculated to verify inclusion/exclusion criteria. At this visit, blood samples were collected for WBC, liver and renal function tests, CD4+ cell count, and HIV-VL. All medications used by the participants during the two months before enrolment were listed and contraindications and interactions with study drugs were checked.

The trial baseline visit (D1) was 15 days after the screening visit, i.e., 30 days after TB treatment initiation. Participants were randomized to one of the arms and ARV treatment was started. Blood samples were collected for WBC, liver and renal function tests, CD4+ cell counts, and HIV-VL. Additionally, a rapid pregnancy test in urine was performed among women.

Participants returned on days D30, D60, D90, D120, D150, and D180 after the beginning of ARV treatment (D1) for medical evaluation that included anamnesis, physical examination, and a thorough inquiry about AE. In all these visits, blood samples were collected for WBC, liver and renal function tests, CD4+ cell count, and HIV-VL.

Sputum smear was collected monthly among patients with pulmonary TB and chest radiographs performed at follow-up visits on D60, D120, and D150. External data monitoring was provided by Fiocruz clinical trial platform.

### 2.7. Sample Size

To calculate the number of participants in the trial, a scenario was constructed to detect differences between the main outcome (proportion of undetectable HIV-VL). The confidence level (a-*α*) was set at 0.95 (z= 1.96), the statistical power (1-*β*) at 0.80 (z= 0.84). The proportion of undetectable HIV-VL with EFV 600 mg was set at 55%, based on the previous cohort at INI (unpublished data), and the expected response rate to EFV 800 mg was set at 75%, as assessed in HIV clinical trials without TB [5]. A 20% percentage difference in response was accepted as clinically relevant by the authors and would justify, according to the Ministry of Health, recommendation to change the national guidelines. The estimated number of participants in each treatment arm was 89.

### 2.8. Data Analysis

All analyses were conducted with R-project software, version 3.3.2 using rms, Hmisc, and epiDisplay packages. Baseline characteristics were compared in both arms to check random allocation. Continuous variables were shown as mean and standard deviation if Gaussian distribution could not be rejected and as median and interquartile range otherwise. Efficacy of treatment was assessed using crude risks, relative risks, and their respective 95% confidence intervals (CI) on D180 using both intention-to-treat and per protocol approaches. The Kaplan-Meier estimator, the log-rank test, and the hazard ratio (HR) were used to compare the intervention and observation groups using time to the first undetectable HIV-VL as outcome (intention-to-treat approach). Crude and correlation-adjusted HIV-VL trajectories were also compared by their confidence bands. The adjusted trajectory was obtained through least squares estimates of the coefficients of a restricted cubic spline function in time after adjusting for subject effects. Then the fit was bootstrapped 500 times, treating time and subject as random variables. Samples were taken jointly from the time, subject, and response vectors to obtain unconditional distributions. Tibshirani's method [17] was used to obtain simultaneous confidence sets for the set of coefficients of the spline as well as the average intercept parameter (over subjects). The same trajectory analyses were conducted in a subset of participants with more than 50 kg and also stratified by high (≥ 5 log) and low (< 5 log) initial HIV-VL.

The proportion of AE (specific events over total events) according to classes, type, and intensity was compared using either the Fisher exact test or Pearson *X*^2^ for contingency tables. WHO-ART system-organ classes [15] and specific events (either expected or nonexpected event) and their intensity and seriousness classifications were compared between study arms. The same intention-to-treat approach with Kaplan-Meier estimator, the log-rank test, and the HR were used to compare the study arms using time to the first treatment interruption due to AE as outcome.

### 2.9. Ethics and Role of the Funding Source

The study was approved by independent ethics committees of the trial's sites and only patients signing an IC were included. This protocol was funded by the SDT/AIDS Program (UNODC) and VPPLR (PDTSP* ensaios clínicos*) — FIOCRUZ. The funding institutions are not responsible for the content of this manuscript. Termination of trial was approved by the centres' ethical committees.

## 3. Results

**Figure 2:**
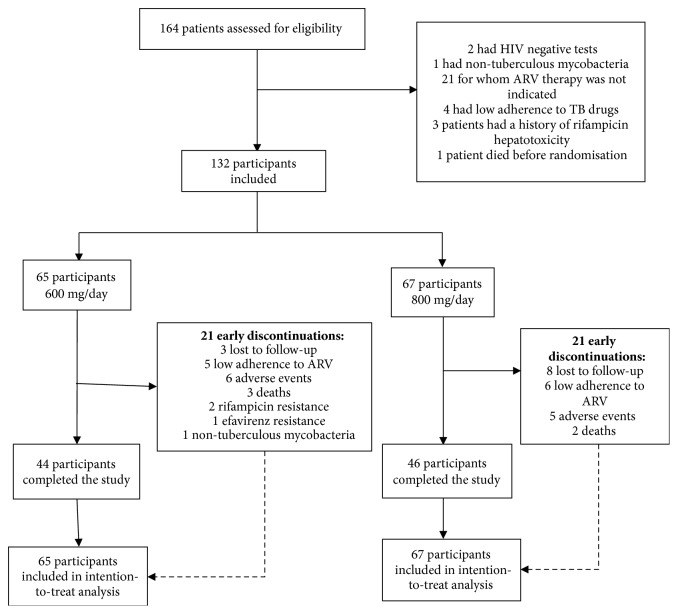
Flowchart representing trial population.

Between April 24, 2006 and March 19, 2012, 164 patients were screened. A total of 132 participants were randomized (65 to comparison arm and 67 to intervention arm), 91 from INI/Fiocruz, 34 from NDI/UFES, and 7 from SCM. In both arms, 21 participants discontinued treatment (Figure 2).

Baseline characteristics and surrogate markers of TB-HIV patients were generally similar among intervention and comparison groups. The trial population was composed predominantly by young male patients with mean weight of 57 kg and with disseminated or pulmonary TB. The majority had CD4+ cell counts below 200 cells/mm^3^ and high HIV-VL levels (Table 1).

### 3.1. Efficacy Outcomes

Proportion of undetectable HIV-VL in the intention-to-treat analysis was 64.6% (95% CI= 52.5%-75.1%) in the comparison arm and 62.7% (95% CI= 50.7%-73.3%) in the intervention arm resulting in a relative risk (EFV800/EFV600) of 0.97 (95% CI= 0.75–1.3). In the per protocol analysis, proportion of undetectable HIV-VL in the comparison arm was 95.5% (95% CI= 89.9%-98.7%) and 91.3% (95% CI= 79.7%-96.6%) in the intervention arm. The relative risk (EFV800/EFV600) as per protocol was 0.96 (95% CI= 0.86-1.1).

**Figure 3:**
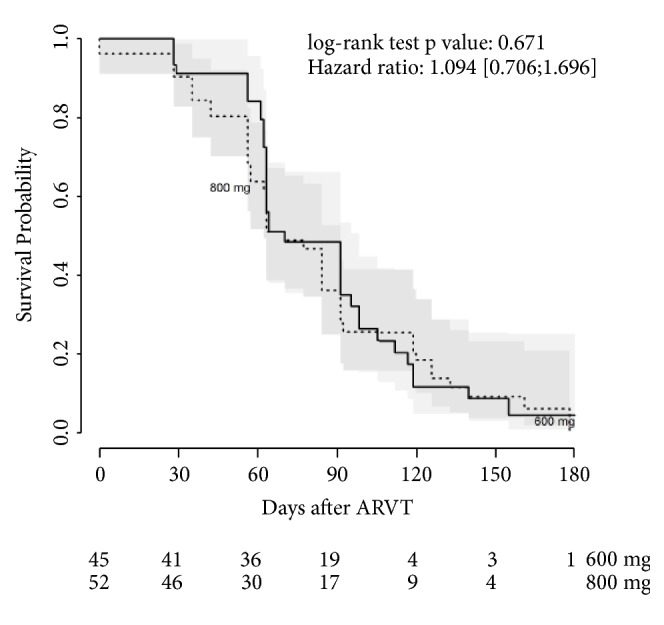
Kaplan-Meier survival analysis with time to achieve first undetectable viral load as outcome in 123 participants using EFV and RIF regimens, stratified by trial arm (800mg EFV-regimen or 600mg EFV-regimen). ARVT: antiretroviral therapy. The dark shade area shows EFV800's survival curve confidence band and the light one shows EFV600's survival curve confidence band (CI=95%). Hazard ratios were calculated as hazard of EFV800 over hazard of EFV600.

The HR (EFV800/EFV600) of time to attain undetectable HIV-VL was 0.93 (95% CI= 0.62-1.4). Median time to this event was similar: 63 (95% CI= 63-91) days in the comparison arm and 70 (95% CI= 63-91) days in the intervention arm. Confidence bands from Kaplan-Meier curves overlapped during all the observed period (Figure 3). The cumulative risk of having undetectable HIV-VL by Kaplan-Meier analyses was 0.90 (95% CI= 0.76-0.96) in the comparison arm and 0.90 (95% CI= 0.77-0.96) in the intervention arm.

**Figure 4:**
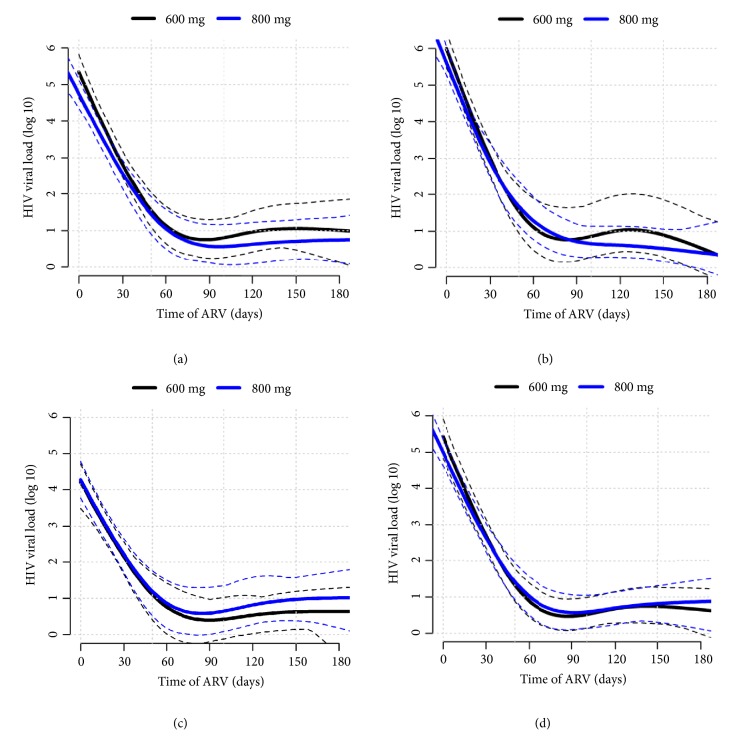
HIV viral load (log 10) trajectories over time during antiretroviral treatment outcome in 123 participants using EFV and RIF regimens according to trial arm (800mg EFV-regimen or 600mg EFV-regimen). Full lines are correlation-adjusted trajectories. Dotted lines are the confidence bands (CI=95%). (a) HIV viral load (log 10) trajectories over time. (b) HIV viral load trajectories over time for initial HIV viral load (log 10) ≥ 5. (c) HIV viral load trajectories over time for initial HIV viral load (log 10) < 5. (d) HIV viral load (log 10) trajectories over time in patients with >50kg (EFV600 N= 50 / EFV600 N= 48).

HIV-VL trajectories 95% bands overlapped across all time periods in the overall study population (Figure 4(a)). It also occurred in both stratified analyses: in HIV-VL ≥ 5 log (Figure 4(b)) and < 5 log (Figure 4(c)) strata, and ≥ 50 kg of weight stratum (Figure 4(d)).

### 3.2. Safety (SSAE) and Tolerance (Minor AE) Outcomes

Three hundred and twenty-two AE were reported: 162 [50.3% (95% CI= 44.8%-55.7%)] in the comparison arm and 160 (49.7% [95% CI 44.3%-55.1%]) in the intervention arm (Table 2). Most (79.6%) events were grade 1 or 2.

Of the 322 AE, 63 (19.6%) were immune or infectious events, 52 (16.2%) were neuropsychiatric, 51 (15.8%) were gastrointestinal, 39 (12.1%) were cutaneous, and 21 (6.5%) were liver disorders. Other, less frequent events (less than 10 records each) were blood, cardiovascular, endocrine, hearing, metabolic, musculoskeletal, neoplasms, reproductive, urinary, vascular, and vision disorders.

**Figure 5:**
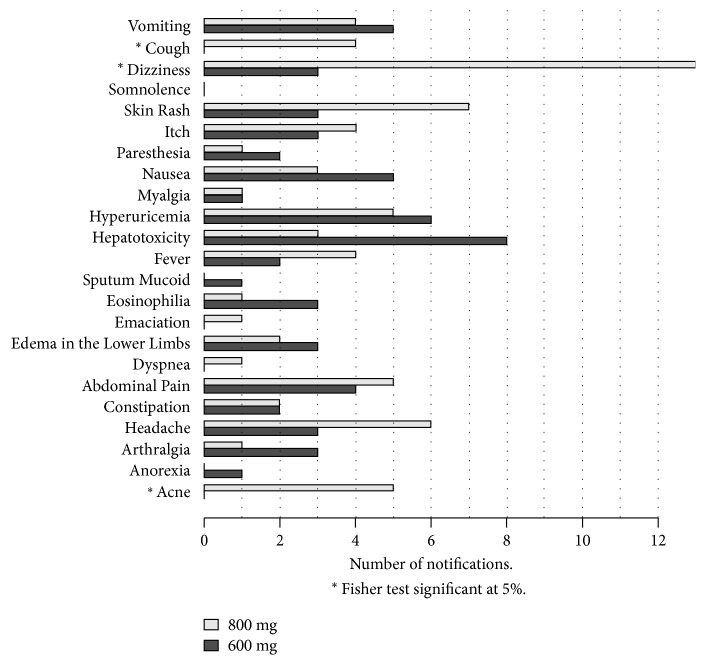
Observed adverse events in 123 participants using EFV and RIF regimens according to trial arm (800mg EFV-regimen or 600mg EFV-regimen).

Regarding the specific type of AE, only cough, acne, and dizziness were significantly more frequent in the intervention arm than in the comparison arm (Figure 5).

SSAE events were homogeneously distributed between both arms. Most of these events were immune, infectious, and gastrointestinal disorders (Table 3). The proportion of SSAE was 19.1 (95% CI= 13.8%-25.8%) in the comparison arm and 25.0% (95% CI= 18.9%-33.2%) in the intervention arm. Relative risk (EFV800/EFV600) for SSAE was 1.3 (95% CI= 0.86-2.0).

Early withdrawal attributable to EA occurred among six participants in the comparison arm and five participants in the intervention arm. The causes of withdrawals in the comparison arm were cutaneous rash and hepatotoxicity, nausea, hepatotoxicity, cutaneous rash plus gastric intolerance, and cutaneous rash and pruritus, while in the intervention arm causes were hallucination plus dizziness, dizziness and insomnia, hepatotoxicity, skin rash and pruritus and rash, with one event each.

**Figure 6:**
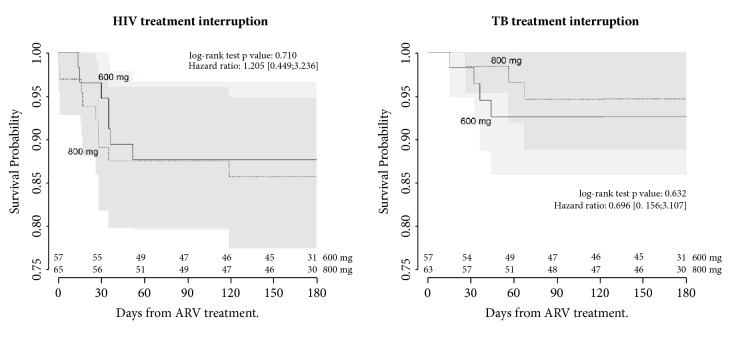
Kaplan-Meier survival analysis with the first treatments interruption as outcome (HIV and tuberculosis) according to trial arm. Dark shade area shows EFV800's survival curve confidence band and light shade shows EFV600's survival curve confidence band (CI=95%). Hazard ratios were calculated as hazard of EFV800 over hazard of EFV600.

Temporary or permanent discontinuation of treatment (early withdrawal) was required for some SSAE. The hazard of EFV discontinuation due to SSAE was similar in both arms (HR: 1.2, 95%CI= 0.45-3.2) as was for RIF discontinuation (HR: 0.70, 95%CI= 0.16-3.1). Confidence bands from Kaplan-Meier curves overlapped throughout the study period, confirming the absence of difference between arms (Figure 6). Five deaths (3.8%) occurred during the trial; the deaths were balanced between both arms and all deaths were due to TB severity.

## 4. Discussion

In the current trial, designed to detect differences in efficacy and safety of two different EFV dosages when used in association with RIF, no differences between daily doses of 600 mg or 800 mg were found regarding both efficacy (per protocol and intention-to-treat analyses) and proportion of SSAE. Likewise, treatment interruption attributable to SSAE (either HIV or TB treatment) was infrequent and similar in both arms. Other endpoints were also similar in both study arms. However, minor AE were more frequent with 800 mg EFV. Altogether, these findings do not support that the dose of EFV should be increased to 800 mg for TB-HIV patients using RIF-containing regimens.

The overall absolute effect (intention-to-treat analysis) of 600 mg EFV was lower in our study than in a trial with non-TB patients in an African country, where 77% had undetectable HIV-VL at 180 days of follow-up [18]. In the REFLAT study [19], it was also conducted in Brazilian TB-HIV patients to compare efficacy of EFV 600mg and raltegravir using rifampicin, and EFV efficacy was similar to this trial. Thus, EFV may have less efficacy when used simultaneously with RIF, regardless of its daily dosage. It is beyond the scope of the current trial to discuss reasons for a smaller effectiveness among Brazilian patients, but advanced disease at baseline and socioeconomic characteristics may play a role in such findings.

Pharmacokinetics studies had shown a decrease in the area under the curve of EFV when used with RIF [8–10]. Therefore, authors have suggested the use of higher doses of EFV in this situation. Clinical evidence from one observational cohort in Africa [20] and an open-labelled randomized study in Thailand [11] did not support this recommendation. Our results corroborate, with better-quality evidence, findings from these previous clinical studies. Because these studies [11, 21] had suggested that only patients weighting over 50 kg or with higher HIV-VL could benefit from higher doses of EFV, we conducted subgroup analyses in patients with these characteristics. We found no advantage in increasing EFV dosage even in these strata.

EFV is, in general, a well-tolerated drug used in fixed-dose combination with other ARV agents in most developing countries worldwide [12]. The most common AE associated with EFV use are neuropsychiatric manifestations and skin rash [12]. In the current trial, we also found that those were the most frequent AE, but we found no significant differences in the frequency of SSAE and time to treatment interruption in both groups. Moreover, minor (grade 1 and 2) AE such as acne, cough, and dizziness were observed more frequently in the EFV800 arm, a finding previously reported [22]. Cough is a common symptom/sign in TB patients, and persistence of cough due to treatment can mislead both healthcare providers and patients. Hepatotoxicity, a frequently reported AE from RIF when used with EFV [23], because of the increased bioavailability of RIF, was infrequent and was equally distributed between both arms. Although symptoms from minor AE were mild and transient (data not shown), altogether, findings from safety and similar effectiveness favour the use of 600 mg of EFV with RIF-containing regimens.

It is well established that genetics may influence drug metabolism resulting in variable clinical response. Pharmacokinetics and pharmacogenetics analyses have predicted virologic endpoints to EFV in countries with homogeneous black population [24]. The population of the current study sites is predominantly Afro- and European-descendant, 25% of participants of black race, which could have had implications in our findings. Unfortunately, because of budget restraints, we were not able to perform pharmacokinetics or pharmacogenetics analyses, and sample size was insufficient for subgroup analyses.

Another limitation was the long time to recruit TB-HIV patients and the insufficient recruitment, due to the organization of the Brazilian public health service, since TB as well as HIV patients are assisted in primary care facilities close to their residence. Initially, INI, a tertiary care hospital, was the only recruitment site. To overcome the low recruitment, other sites were invited to join the trial. However, differences between groups were too small and a much larger sample size than calculated would be needed to show any significant difference. Thus, the absence of evidence of efficacy could be due to the reduced power of the study with the current sample. However, trajectories of HIV-VL were absolutely overlapping, and the authors do not believe that increasing the sample size would change the results. The power to detect a significant effect regarding undetectable HIV-VL with the risk ratio actually found (0.97) is indeed very low (2.7%). It would be necessary to include approximately 20,330 participants to detect a 5% significance for this effect, which would be unachievable even in a multicentre trial. Therefore, there was an understanding among authors that reaching the initially planned sample size of 178 would not change the current results interpretation because the low power, subgroup analyses should also be interpreted with caution.

Additionally, observation bias cannot be excluded since both patients and physicians were unblinded for the arm. Regarding safety, this bias would result from an excess of reports of SSAE in the intervention arm, which was not observed. Since no differences were found regarding both efficacy and safety (SSAE), bias is unlikely. Finally, the long follow-up period could have resulted in potential external interference not explored in the trial.

Our study also has a few strengths. This was a pragmatic trial, sponsored by the Brazilian Ministry of Health, with no influence of private laboratories or other market partners. Thus, findings can be expected to be reproducible in routine conditions. The evidence presented here does not support previous pharmacokinetics suggestions that EFV dose should be increased when prescribed with RIF in TB-HIV patients to increase its efficacy. Although there is no evidence that SSAE and interruption of treatment due to SSAE are more frequent with a higher EFV dose, the slight increase of specific AE among participants taking 800 mg EFV and the lack of difference in efficacy favour the use of 600 mg of EFV even when associated with RIF-containing regimens.

## Figures and Tables

**Table 1:** Baseline and clinical characteristics of the trial population according to trial arm (800mg EFV-regimen or 600mg EFV-regimen). ALT= alanine transaminase. AST= aspartate transaminase. HBsAg= hepatitis B surface antigen. Anti-HCV= hepatitis C antibody. HIV= Human Immunodeficiency Virus. TB=tuberculosis.

	**EFV600**	**EFV800**	**Total**
**Total**	**65**	**67**	**132**
**Age (years)**			
mean (SD)	38.3 (9.9)	37.3 (9.8)	37.8 (9.8)
**Sex**			
Male	51 (78.5%)	57 (86.4%)	108 (82.4%)
Female	14 (21.5%)	9 (13.6%)	23 (17.6%)
**Ethnicity**			
White	24 (36.9%)	21 (31.8%)	45 (34.4%)
Black	8 (12.3%)	23 (34.8%)	31 (23.7%)
Mixed	33 (50.8%)	22 (33.3%)	55 (42.0%)
**Weight(Kg)**			
median (IQR)	57.0 (50.9, 62.3)	57.4 (51.0, 63.0)	57.0 (50.9, 62.9)
**Weight (Kg)**			
≤ 50	15 (23.1%)	13 (21.3%)	28 (22.2%)
> 50	50 (76.9%)	48 (78.7%)	98 (77.8%)
**Tuberculosis Clinical Forms**			
Pulmonary	34 (52.3%)	27 (40.3%)	61 (46.2%)
Extrapulmonary	5 (7.7%)	5 (7.5%)	10 (7.6%)
Ignored	1 (1.5%)	2 (3.0%)	3 (2.3%)
Disseminated	25 (38.5%)	33 (49.2%)	58 (43.9%)
**Karnofsky Index**			
median (IQR)	90 (80.0, 90.0)	90 (80.0, 90.0)	90 (80.0, 90.0)
**AST (U/L)**			
median (IQR)	38.0 (28.5, 50.8)	40.0 (30.0, 48.0)	39.0 (30.0, 49.0)
**ALT (U/L)**			
median (IQR)	38.0 (28.2, 47.0)	37.0 (27.0, 48.0)	37.0 (27.0, 48.0)
**Total Bilirubin (mg/dL)**			
median (IQR)	0.4 (0.3, 0.6)	0.4 (0.3, 0.5)	0.4 (0.3, 0.6)
**CD4+ (cell/mm** ^**3**^ **)**			
≤ 200	41 (66.1%)	45 (68.2%)	86 (67.2%)
> 200	21 (33.9%)	21 (31.8%)	42 (32.8%)
**HIV viral load (log)**			
< 5	21 (35.0%)	27 (43.5%)	48 (39.3%)
≥ 5	39 (65.0%)	35 (56.5%)	74 (60.7%)
**Previous TB treatment**			
No	55 (91.7%)	55 (88.7%)	110 (90.2%)
Yes	5 (8.3%)	7 (11.3%)	12 (9.8%)
**HBsAg**			
Negative	44 (68.8%)	41 (64.1%)	85 (66.4%)
Positive	2 (3.1%)	6 (9.4%)	8 (6.2%)
Not done	18 (28.1%)	17 (26.6%)	35 (27.3%)
**Anti-HCV**			
Negative	52 (81.2%)	51 (79.7%)	103 (80.5%)
Positive	1 (1.6%)	4 (6.2%)	5 (3.9%)
Not done	11 (17.2%)	9 (14.1%)	20 (15.6%)

**Table 2:** Reported adverse events severity according to trial arm. All adverse events reported throughout the study according to severity.

	**EFV600**	**EFV800**	**Total**	**Test statistics**	**P value**
**Total**	**162**	**160**	**322**	Chi square (3 df) = 2.47	0.48
Grade 1	75 (46.3%)	61 (38.1%)	136 (42.2%)		
Grade 2	54 (33.3%)	58 (36.2%)	112 (34.8%)		
Grade 3	20 (12.3%)	24 (15.0%)	44 (13.7%)		
Grade 4	13 (8.0%)	17 (10.6%)	30 (9.3%)		

**Table 3:** Prevalence of severe and serious adverse events according to trial arm. Adverse events grades 3, 4 or serious adverse events were grouped according to WHO-ART guidelines and compared if there was a difference between the two arms.

	**EFV600**	**EFV800**	**Total**	**Test stat.**	**P value**
**Total**	**31**	**40**	**71**		

**WHO-ART Classification**					
Blood disorders	1/31 (3.2%)	0/40 (0.0%)	1/71 (1.4%)	Fisher's exact test	0.44
Cardiovascular disorders	2/31 (6.5%)	4/40 (10.0%)	6/71 (8.5%)	Fisher's exact test	0.69
Gastrointestinal disorders	6/31 (19.4%)	6/40 (15.0%)	12/71 (16.9%)	Chi square (1 df) = 0.03	0.87
Immune disorders and infections	10/31 (32.3%)	8/40 (20.0%)	18/71 (25.4%)	Chi square (1 df) = 0.81	0.37
Liver and biliary disorders	4/31 (12.9%)	5/40 (12.5%)	9/71 (12.7%)	Fisher's exact test	1.00
Metabolic and nutritional disorders	0/31 (0.0%)	1/40 (2.5%)	1/71 (1.4%)	Fisher's exact test	1.00
Musculoskeletal disorders	2/31 (6.5%)	1/40 (2.5%)	3/71 (4.2%)	Fisher's exact test	0.58
Neurological disorders	1/31 (3.2%)	6/40 (15.0%)	7/71 (9.9%)	Fisher's exact test	0.13
Psychiatric disorders	0/31 (0.0%)	3/40 (7.5%)	3/71 (4.2%)	Fisher's exact test	0.25
Respiratory disorders	0/31 (0.0%)	1/40 (2.5%)	1/71 (1.4%)	Fisher's exact test	1.00
Skin and appendages disorders	5/31 (16.1%)	2/40 (5.0%)	7/71 (9.9%)	Fisher's exact test	0.23
Urinary tract disorders	0/31 (0.0%)	1/40 (2.5%)	1/71 (1.4%)	Fisher's exact test	1.00
Vascular, bleeding, and clotting disorders	0/31 (0.0%)	2/40 (5.0%)	2/71 (2.8%)	Fisher's exact test	0.50

**Adverse Event Severity**				Fisher's exact test	0.92
Grade 2	1 (3.2%)	2 (5.0%)	3 (4.2%)		
Grade 3	20 (64.5%)	24 (60.0%)	44 (62.0%)		
Grade 4	10 (32.3%)	14 (35.0%)	24 (33.8%)		

**Serious Adverse Event Prevalence**	12/31 (38.7%)	16/40 (40.0%)	28/71 (39.4%)	Chi square (1 df) = 0	1.00

## Data Availability

The data used to support the findings of this study are available from the corresponding author upon request.

## References

[1] Coelho L., Cardoso S. W., Amancio R. T. (2014). Trends in AIDS-Defining Opportunistic Illnesses Incidence over 25 Years in Rio de Janeiro, Brazil. *PLoS ONE*.

[2] World Health Organization (2016). *Global tuberculosis report 2016*.

[3] World Health Organization (2010). *Stop TB Initiative (World Health Organization). Treatment of tuberculosis: guidelines*.

[4] Zumla A., Nahid P., Cole S. T. (2013). Advances in the development of new tuberculosis drugs and treatment regimens. *Nature Reviews Drug Discovery*.

[5] Staszewski S., Morales-Ramirez J., Tashima K. T. (1999). Efavirenz plus zidovudine and lamivudine, efavirenz plus indinavir, and indinavir plus zidovudine and lamivudine in the treatment of HIV-1 infection in adults. *The New England Journal of Medicine*.

[6] World Health Organization (2013). *Consolidated guidelines on the use of antiretroviral drugs for treating and preventing HIV infection: recommendations for a public health approach*.

[7] Liu J., Chan-Tack K. M., Jadhav P. (2014). Why did the FDA approve efavirenz 800 mg when co-administered with rifampin?. *International Journal of Clinical Pharmacology and Therapeutics*.

[8] Lopez-Cortes L. F., Ruiz-Valderas R., Pompyo V. (2002). Pharmacokinetic interactions between efavirenz and rifampicin in HIV-infected patients with tuberculosis. *Clinical Pharmacokinetics*.

[9] Matteelli A., Regazzi M., Villani P. (2007). Multiple-dose pharmacokinetics of Efavirenz with and without the use of Rifampicin in HIV-positive patients. *Current HIV Research*.

[10] Yenny, Nafrialdi, Djoerban Z., Setiabudy R. (2011). Pharmacokinetic interaction between efavirenz and rifampicin in healthy volunteers. *International Journal of Clinical Pharmacology and Therapeutics*.

[11] Manosuthi W., Sungkanuparph S., Thakkinstian A. (2005). Efavirenz levels and 24-week efficacy in HIV-infected patients with tuberculosis receiving highly active antiretroviral therapy and rifampicin. *AIDS*.

[12] (1999). *Stocrin [package insert]*.

[13] (2011). *Manual de recomendações para o controle da tuberculose no Brasil. Brasília/DF: Ministério da Saúde: Secretaria de Vigilância em Saúde*.

[14] (2002). *Brazil, Coordenação Nacional de DST e Aids. Atualização das recomendações para tratamento da co-infecção HIV/tuberculose em adultos e adolescentes*.

[15] (2015). *Uppsala Monitoring Center. The WHO-ART Adverse Reaction Terminology*.

[16] *U.S. Department of Health and Human Services, National Institutes of Health, National Institute of Allergy and Infectious Diseases, Division of AIDS. Division of AIDS Table for Grading the Severity of Adult and Pediatric Adverse Events*.

[17] Tibshirani R., Knight K. (August 1997). Model search and inference by bootstrap bumping. http://www-stat.stanford.edu/~tibs.

[18] Landman R., Schiemann R., Thiam S. (2003). Once-a-day highly active antiretroviral therapy in treatment-naive HIV-1-infected adults in Senegal. *AIDS*.

[19] Grinsztejn B., De Castro N., Arnold V. (2014). Raltegravir for the treatment of patients co-infected with HIV and tuberculosis (ANRS 12 180 Reflate TB): A multicentre, phase 2, non-comparative, open-label, randomised trial. *The Lancet Infectious Diseases*.

[20] Orrell C., Cohen K., Conradie F. (2011). Efavirenz and rifampicin in the South African context: Is there a need to dose-increase efavirenz with concurrent rifampicin therapy?. *Antiviral Therapy*.

[21] Manosuthi W., Mankatitham W., Lueangniyomkul A., Chimsuntorn S., Sungkanuparph S. (2008). Standard-dose efavirenz vs. standard-dose nevirapine in antiretroviral regimens among HIV-1 and tuberculosis co-infected patients who received rifampicin. *HIV Medicine*.

[22] McIlleron H., Meintjes G., Burman W. J., Maartens G. (2007). Complications of antiretroviral therapy in patients with tuberculosis: Drug interactions, toxicity, and immune reconstitution inflammatory syndrome. *The Journal of Infectious Diseases*.

[23] Puri P., Kaur N., Pathania S., Kumar S., Sharma P. K., Sashindran V. K. (2017). Antitubercular therapy induced liver function tests abnormalities in human immunodeficiency virus infected individuals. *Medical Journal Armed Forces India*.

[24] Stöhr W., Back D., Dunn D. (2008). Factors influencing efavirenz and nevirapine plasma concentration: Effect of ethnicity, weight and co-medication. *Antiviral Therapy*.

